# SpliceAPP: an interactive web server to predict splicing errors arising from human mutations

**DOI:** 10.1186/s12864-024-10512-x

**Published:** 2024-06-15

**Authors:** Ang-Chu Huang, Jia-Ying Su, Yu-Jen Hung, Hung-Lun Chiang, Yi-Ting Chen, Yen-Tsung Huang, Chen-Hsin Albert Yu, Hsin-Nan Lin, Chien-Ling Lin

**Affiliations:** 1 Institute of Molecular Biology, Academia Sinica, No. 128, Sec. 2, Academia Road, Nangang District, Taipei City, 115014 Taiwan; 2 Genome and Systems Biology Degree Program, Academia Sinica and National Taiwan University, Taipei, Taiwan; 3 Institute of Statistical Science, Academia Sinica, Taipei, Taiwan; 4 Bioinformatics Program, International Graduate Program, Academia Sinica, Taipei, Taiwan; 5 Institute of Biomedical Informatics, National Yang Ming Chiao Tung University, Taipei, Taiwan

**Keywords:** RNA splicing, Human mutations, Splicing variant prediction, LASSO regression

## Abstract

**Background:**

Splicing variants are a major class of pathogenic mutations, with their severity equivalent to nonsense mutations. However, redundant and degenerate splicing signals hinder functional assessments of sequence variations within introns, particularly at branch sites. We have established a massively parallel splicing assay to assess the impact on splicing of 11,191 disease-relevant variants. Based on the experimental results, we then applied regression-based methods to identify factors determining splicing decisions and their respective weights.

**Results:**

Our statistical modeling is highly sensitive, accurately annotating the splicing defects of near-exon intronic variants, outperforming state-of-the-art predictive tools. We have incorporated the algorithm and branchpoint information into a web-based tool, SpliceAPP, to provide an interactive application. This user-friendly website allows users to upload any genetic variants with genome coordinates (e.g., chr15 74,687,208 A G), and the tool will output predictions for splicing error scores and evaluate the impact on nearby splice sites. Additionally, users can query branch site information within the region of interest.

**Conclusions:**

In summary, SpliceAPP represents a pioneering approach to screening pathogenic intronic variants, contributing to the development of precision medicine. It also facilitates the annotation of splicing motifs. SpliceAPP is freely accessible using the link https://bc.imb.sinica.edu.tw/SpliceAPP. Source code can be downloaded at https://github.com/hsinnan75/SpliceAPP.

## Background

### Complexities of splicing regulations

RNA splicing is a fundamental cellular process responsible for connecting exons for translation and removing introns for nucleic acid recycling. It facilitates RNA export and translation, and is highly regulated in a temporal and spatial manner, contributing to the complexity of an organism’s transcriptome [1]. Splicing signals, including the 5’ splice site (5’ss), branch site, and 3’ splice site (3’ss), play crucial roles in orchestrating this intricate process. Additional elements, such as a polypyrimidine tract downstream of the branch site and an AG dinucleotide exclusion zone, aid in the recognition of the 3’ss [2]. Mechanistically, the 5’ss base pairs with U1 small nuclear RNA (snRNA), and the branch site pairs with the recognition sequence of U2 snRNA [3]. The stabilization of U2 small nucleoproteins (snRNPs) on the branch site is facilitated by the interplay of U2 auxiliary factor 2 and U2 auxiliary factor 1 with the polypyrimidine tract and the 3’ss during 3’ss recognition. This interaction positions the branchpoint for nucleophilic attack on the 5’ss, marking the initial catalytic event of splicing.

## Challenges in splicing variant prediction

The costs of whole-genome sequencing are declining as sequencing technology advances, and the library of human genetic variants is expanding dramatically every day. It is estimated that 10–30% of disease-associated genetic variants affect splicing [4, 5]. Splicing variants may generate deleteriously altered gene products and become potential therapeutic targets. However, predicting redundant and degenerate splicing signals is a major challenge for the functional evaluation of intronic variants. While the 5’ss and 3’ss are well-defined, branch sites, polypyrimidine tracts and many splicing regulatory elements exhibits greater variability in sequence motif and position in higher organisms. Large-scale mapping studies have identified multiple branch sites detected within a given intron [6, 7]. This variability in the splicing motifs poses challenges in interpreting intronic sequence variations near intron-exon boundaries.

## SpliceAPP: transparent splicing variant prediction with LASSO regression

**Fig. 1:**
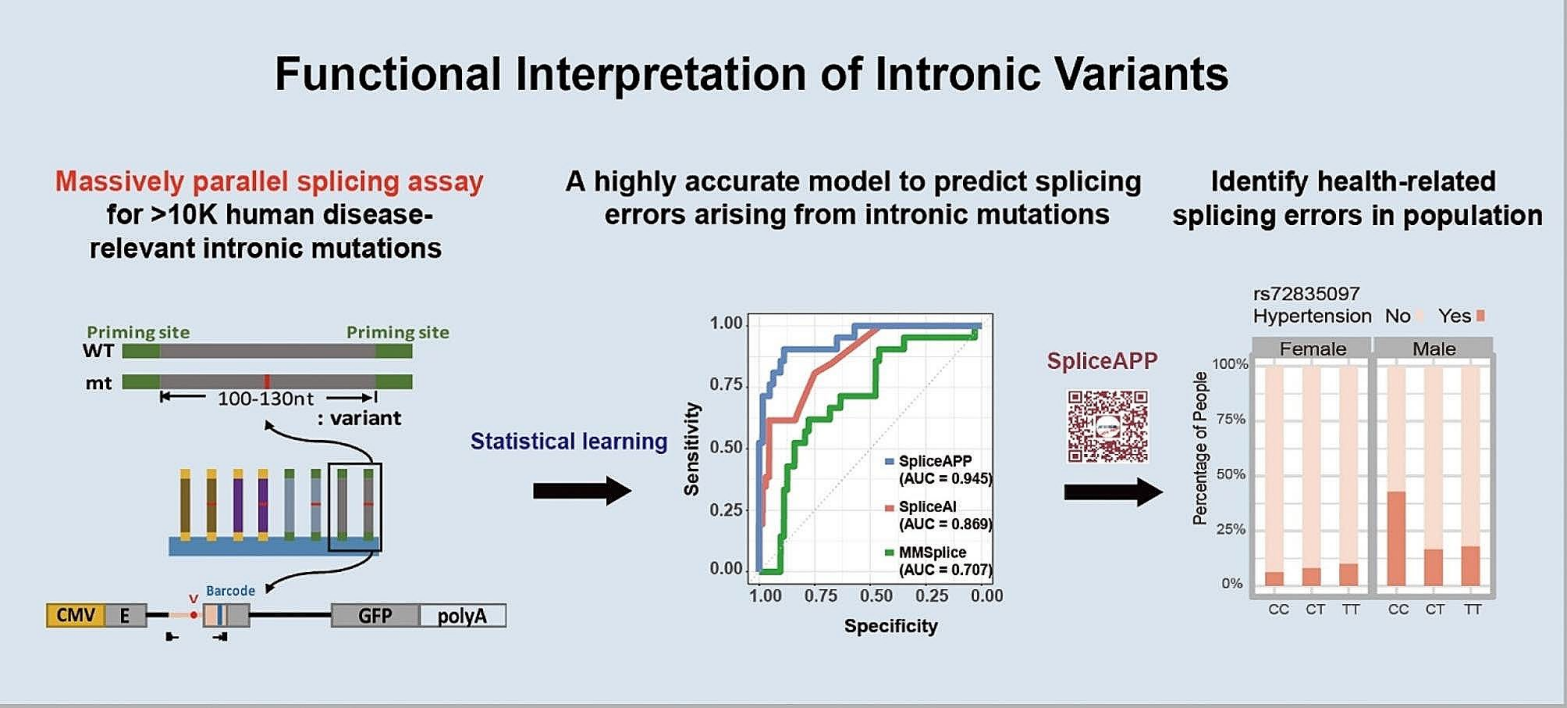
Development of the splicing error predictive model, SpliceAPP, from experimental splicing assays. A total of 11,191 pairs of oligos containing human disease-relevant mutations were synthesized in bulk and ligated into 3-exon splicing minigenes. By contrasting variants with and without a splicing defect, we developed an explanatory and predictive model, SpliceAPP, that classifies splicing variants with high sensitivity and specificity. The algorithm not only explains the mechanism of splicing decisions but is also useful in annotating defects of non-coding variants that may potentially affect human health

Deep-learning has been widely deployed to develop splicing predictive tools capable of interpreting intronic mutations [4, 8, 9]. Nevertheless, those models trained on specifying canonical splice sites from intergenic splice site-like sequence (GT or AG) or alternative splice sites based on flanking sequence perform moderately in terms of detecting splice-altering intronic mutations. This limitation arises from the fact that disease-related mutations often occur within sequences resembling wild-type counterparts, leading to minimal alterations in gene structure scores. Additionally, some predictive models are constrained by their focus solely on predicting splice site and exonic splicing variants [10–12]. Moreover, the inherent limitations of deep learning prevent the establishment of the significance of each input factor, which hampers further model refinement and advancement. Therefore, we established a LASSO regression model from a massively parallel splicing assay on 11,191 human disease-relevant intronic mutations. In this assay, both reference and alternative alleles spanning the splice sites were synthesized in bulk into DNA oligos and then ligated into three exon-containing splicing minigenes. These minigenes, equipped with CMV promoters and polyadenylation signals, were transfected into HEK293T cells for expression and splicing. Subsequently, the splicing outcomes were resolved by amplicon sequencing using primers on the flanking common exons. The difference of splicing efficiency between the reference and alternative allele pairs was determined by Fisher’s exact test **(**Fig. 1**)**. By comparing variants with or without a splicing effect, we identified factors that influence the splicing decision. The LASSO penalty was selected by tuning toward the minimal deviance for regression. Hence, we used the following formula to determine the coefficient of influential features in splicing decisions:

$$\text{L}\text{o}\text{g}\frac{\text{P}({\text{Y}}_{\text{i}}=1)}{1-\text{P}({\text{Y}}_{\text{i}}=1)}={{\beta }}_{0}+\sum _{\text{j}=1}^{\text{p}}{{\beta }}_{\text{j}}{\text{X}}_{\text{j}\text{i}}$$

where *Y*_*i*_=1 is the *i*^th^ alternative sequence that significantly affects splicing, and *X*_*ji*_ is the feature *j* in the *i*^th^ sequence.

**Fig. 2:**
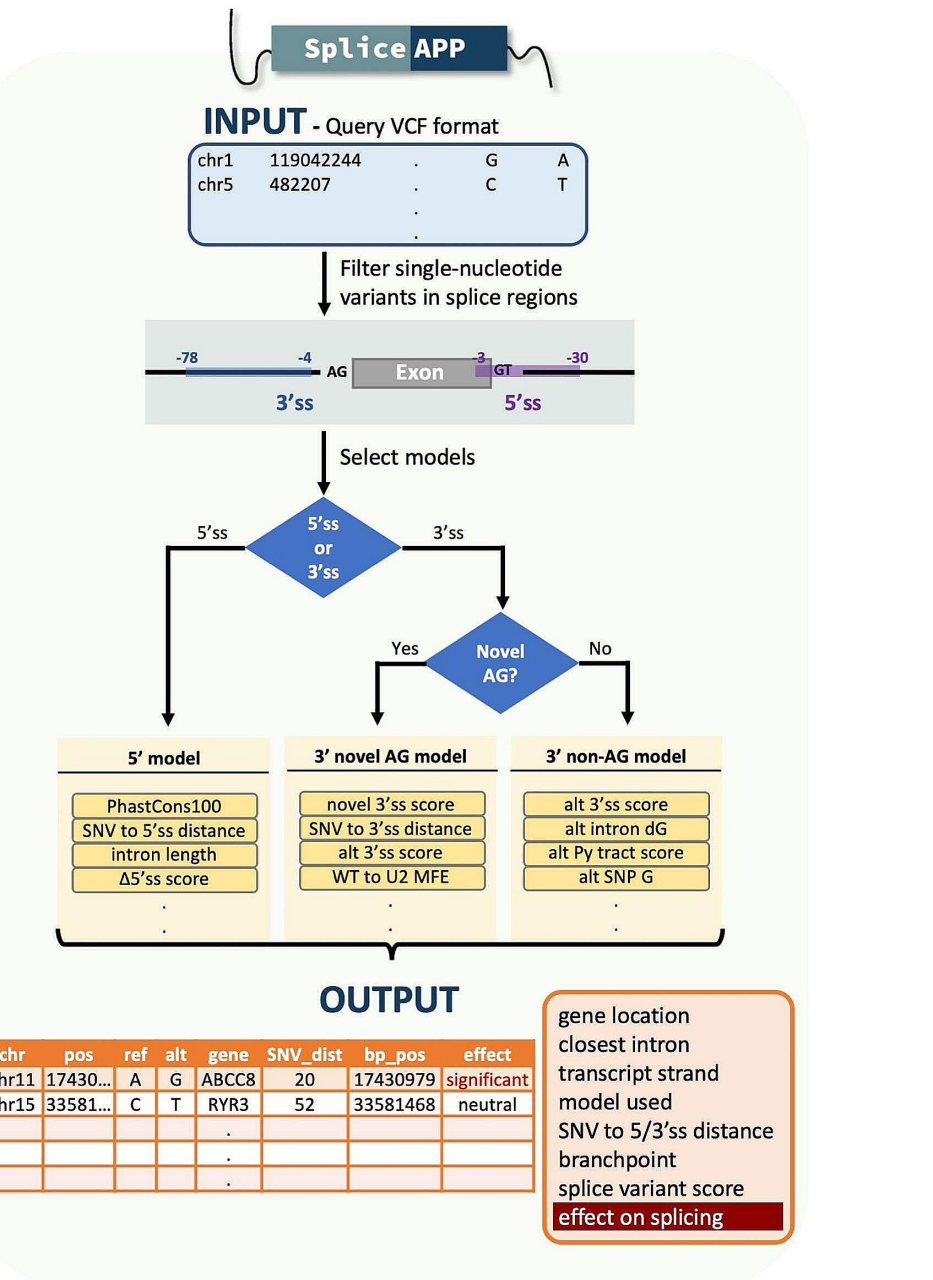
The workflow of SpliceAPP. The tool accepts variant descriptions in Variant Call Format (VCF) and filters out variants not in the splice region and indels. Based on the variants’ position, it is directed to either 5’ splice site (5’ss) or 3’ splice site (3’ss) models. The 3’ss models further categorize variants that generate a novel AG (3’ss) into a separate model. Utilizing pre-trained factors, three models assess the impact of the variant. PhasCons100: evolutionary conservation level, mt: mutation or variant, alt: the alternative allele, MFE: minimum free energy, indicating the pairing energy between the wildtype introns and U2 snRNA, dG: folding energy, Py track: polypyrimidine track, SNP: single nucleotide polymorphism. The output includes basic characterization of the variant and its associated gene and intron, along with the classification of splicing variant

Our method not only incorporates primary sequences, but also combines our knowledge of RNA splicing and genomics, converting this knowledge into parameters for statistical learning **(**Fig. 2**)**. For example, we consider evolutionary conservation, structural openness and sequence folding efficiency, all of which are features that cannot be gleaned from primary sequences. These and other features are selected and weighted by LASSO regression as a formula to predict splicing errors. Given that the mechanisms of 3’ and 5’ splice site (ss) recognition are distinct—involving two distinct spliceosomal complexes, i.e., U2 and U1 snRNPs, respectively—we have trained the models of splicing errors separately. Furthermore, the 3’ss is sensitive to competition from intronic 3’ss created by mutations, so variants that generate intronic 3’ss AG dinucleotides are assessed using a unique competition model. Overall, the final predictive models include three modules: 5’ ss, 3’ ss novel-AG, and 3’ ss non-AG [13].

### Identification of RNA splicing errors for precise genetic diagnosis

**Fig. 3:**
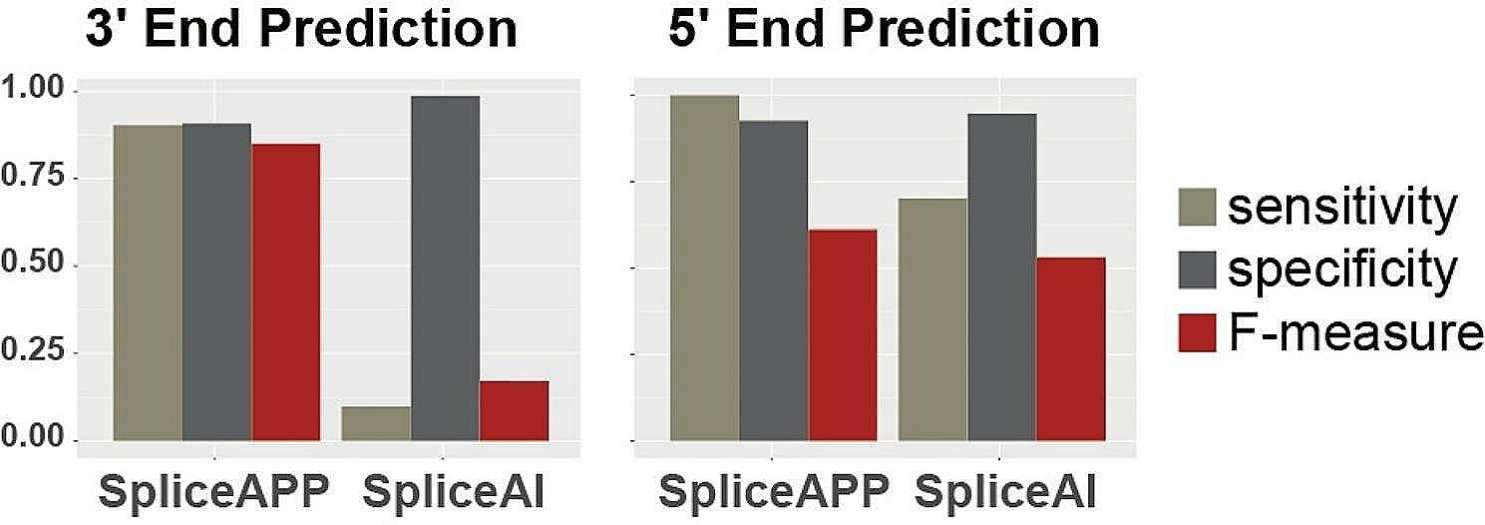
The comparative performance of SpliceAPP and SpliceAI in predicting splicing errors. Sensitivity and specificity measures the algorithm’s ability to correctly classify true positives and negatives [14, 15], respectively. The F-measure, also referred to as F-score, provides an overall assessment of accuracy of an algorithm considering both precision and recall. It is calculated as 2 times the product of precision and recall divided by the sum of precision and recall

Our statistical modeling proved highly sensitive and accurate in annotating the splicing defects of near-exon intronic variants, outperforming the predictive ability of benchmarking predictive tools **(**Fig. 3**)**. In addition, unlike AI (Artificial Intelligence)-based black box models, our factor-based model provides information explaining changes in RNA splicing, and our transparent model can be further refined by experts in all fields. The model established by our lab, which we have named SpliceAPP, is freely available as an interactive web server (https://bc.imb.sinica.edu.tw/SpliceAPP/), providing a platform for precise diagnosis and precision medicine of RNA splicing errors.

## Implementation

Here, we introduce a web application, SpliceAPP (*Splice* *A*lternative *P*rofile-based *P*redictor), which we built to deploy our predictive models for splicing alterations of human intronic variants. SpliceAPP provides a user-friendly interface for querying unknown genetic variants for prediction and searching for the previously predicted variants in the SpliceAPP database. The back-end prediction module of SpliceAPP was developed using C++, and the front-end user interface (UI) has been designed using PHP, HTML, CSS, and JavaScript. SpliceAPP also features a progress bar and an email notification function. Moreover, it facilitates retrieval of information about branchpoints within specific regions. SpliceAPP offers a genome browser equipped with ‘IGV.js’, an interactive genome visualization component. This tool enables users to easily navigate through both predicted and experimental branch site data with the genome features, such as genes and exons. The application is compatible with major web browsers, including Google Chrome, Firefox, Safari, and Internet Explorer. We also provide a standalone version for running SpliceAPP on a local server.

## Results

SpliceAPP ignores genetic variants that are located outside gene regions. If a genetic variant is located between − 3 and + 30 basepairs (bp) from the 5’ end of an intron, it is considered a 5’ variant. If it is located between − 78 and − 4 bp from the 3’ end of an intron, it is a 3’ variant. If a 3’ variant produces a novel AG dinucleotide, it is predicted with the 3’ ss novel-AG model; otherwise, it is 3’ ss non-AG, with predictions performed accordingly. Variants outside of these regions are triaged before prediction analysis but remain in the output table with only the basic gene information.

The model built by our team is highly accurate, outperforming the predictive ability of currently available tools. Specifically, in a validation dataset of 107 intronic 3’ variants (31 splice-altering and 76 with no effect) [14], our model achieved 90.3% sensitivity in terms of detecting splicing variants (90.8% specificity and 90.7% accuracy), outperforming the 9.7% sensitivity of SpliceAI, a splicing prediction tool developed by Illumina. In a separate validation dataset of 314 5’ variants (17 splice-altering and 297 with no effect) [15], our model achieved 100% sensitivity (92.6% specificity and 93.0% accuracy), compared to the 70.1% sensitivity of SpliceAI **(**Fig. 3**)**. The strength of the validation is limited by the size of the available datasets. Nonetheless, SpliceAPP achieves highest accuracy among the tools tested (https://bc.imb.sinica.edu.tw/SpliceAPP/evaluation.html).

**Fig. 4:**
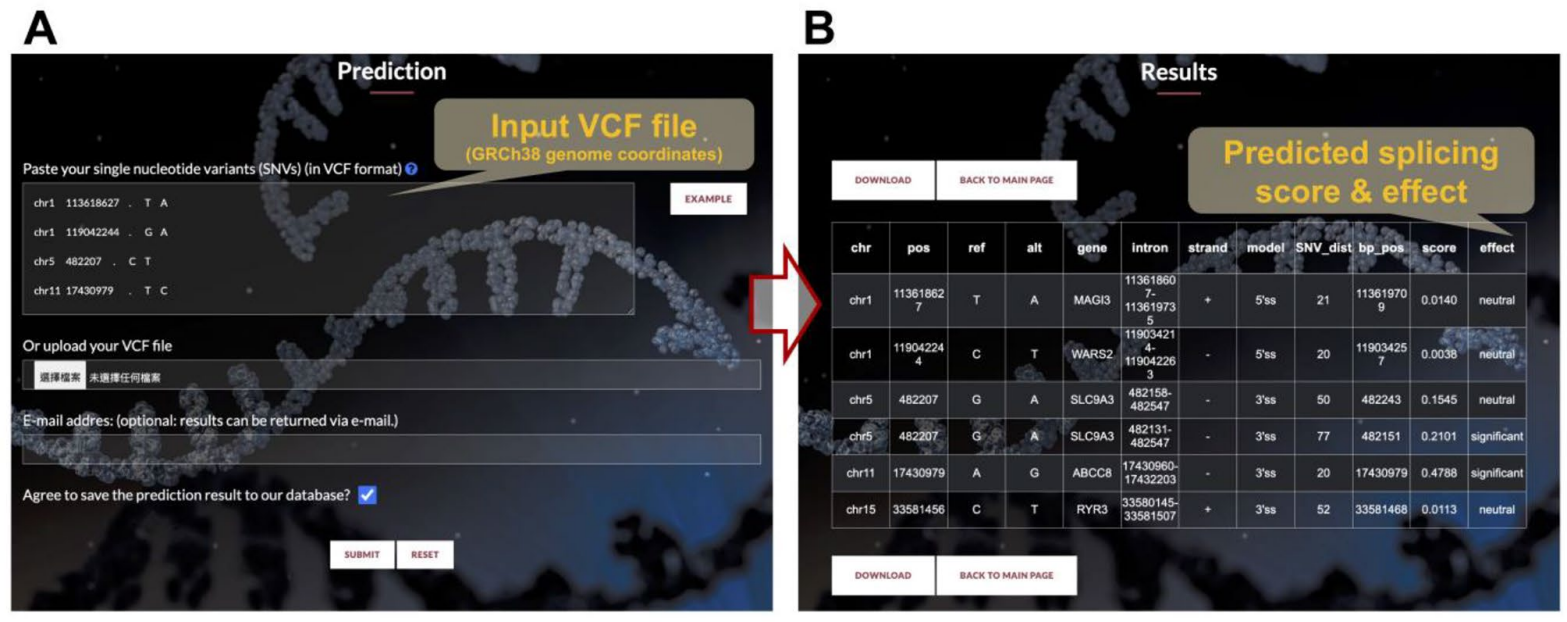
A user-friendly web interface of SpliceAPP. **(A)** SpliceAPP is an interactive web tool that only requires inputs of Variant Call Format (VCF). **(B)** The output will be gene location, genomic coordinates of the closest intron, transcript strand, 3’ or 5’ end predictive model used, distance between the variant to the closest splice site, predicted branchpoint, predicted splice variant score and its effect on splicing of the nearby splice sites

SpliceAPP is a user-friendly website that allows users to upload any variants with chromosomal coordinates and altered allele type information or bulk variants in Variant Call Format (VCF) using human genome assembly GRCh38 (hg38) **(**Fig. 4A**)**. Variants not aligning with the hg38 reference genome or situated outside the computable regions are automatically disregarded from the prediction. The output of SpliceAPP includes the gene where the variant is located, the genomic coordinates of the closest intron, the transcript strand, the 3’ or 5’ end predictive model used, the distance between the variant to the closest splice site, the predicted splice variant score, and its effect on splicing of the nearby splice sites **(**Fig. 4B**)**. The score (0 ~ 1) represents the likelihood of the variant disrupting canonical splicing, and the effect indicates the significance of a splicing error. It is determined by identifying the optimal cutoff point that maximizes both sensitivity and specificity for each independent model. The cutoffs are 0.13 for the 5’ss model, 0.1837238 for the 3’ss AG model, and 0.07784796 for the 3’ss non-AG model. The results can be viewed directly on the webpage or downloaded and then opened in Microsoft Excel. In addition, users can choose to save the predicted results into a SpliceAPP database, serving as a searchable database of collected intronic variants and their predicted splicing effects.

**Fig. 5:**
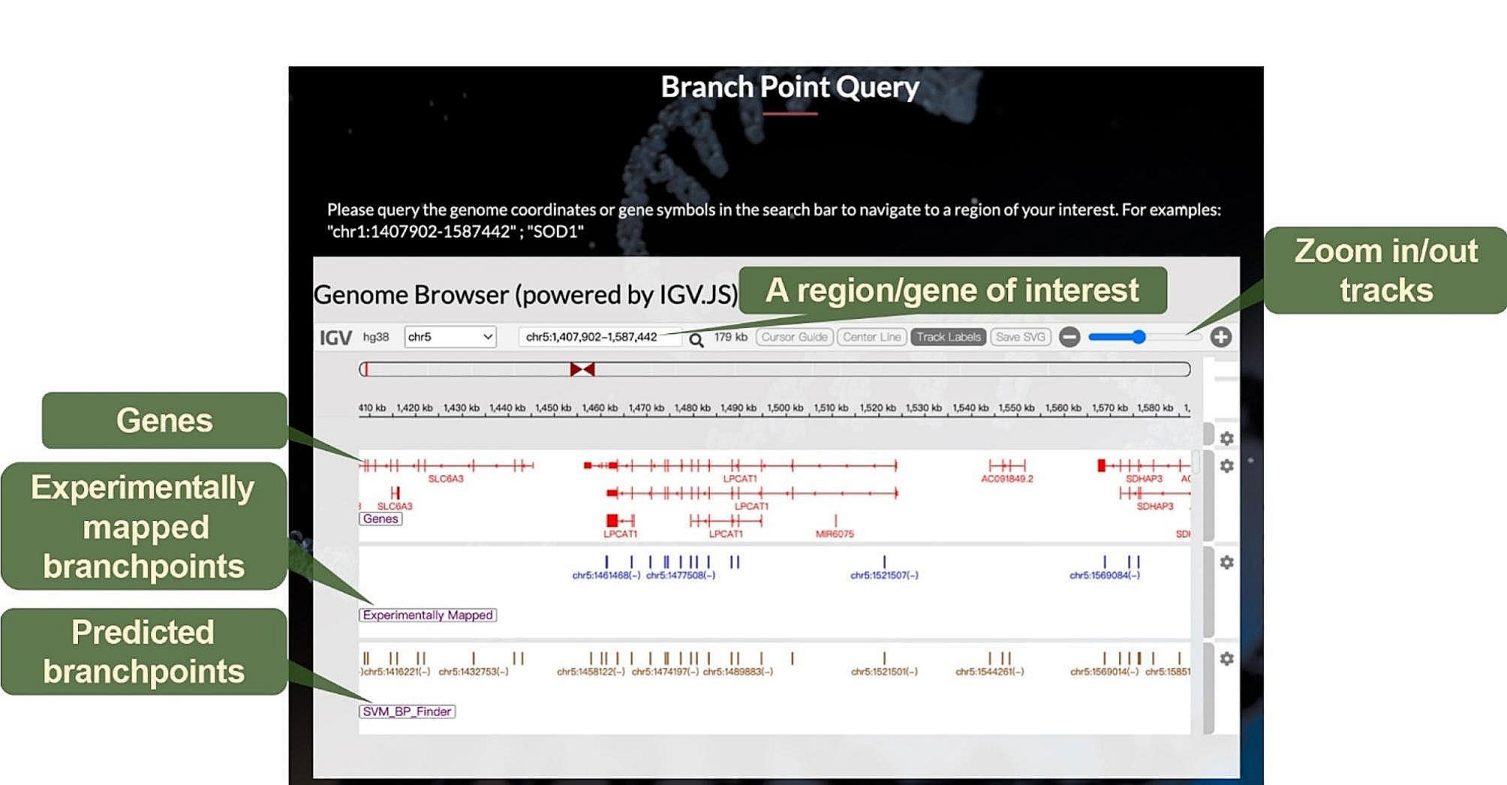
SpliceAPP offers the Branch Point Query function. The Integrative Genomics Viewer (IGV) features predicted branch points identified through SVM-BPfinder, complemented by experimentally derived branch points sourced from prior studies

To provide insights into functional regions in introns, we have compiled information on branchpoint locations discovered in previous studies [6, 7, 16, 17] and integrated the highest-scoring predicted branchpoint sites from SVM-BPfinder [18], presenting them in an Interactive Genomics Viewer (IGV) **(**Fig. 5**)**. Users can input regions of interest and gene names to query if specific areas contain branchpoint information. This user-friendly feature provides accessible and comprehensive information about the functionality of intronic regions.

## Discussion

**Fig. 6:**
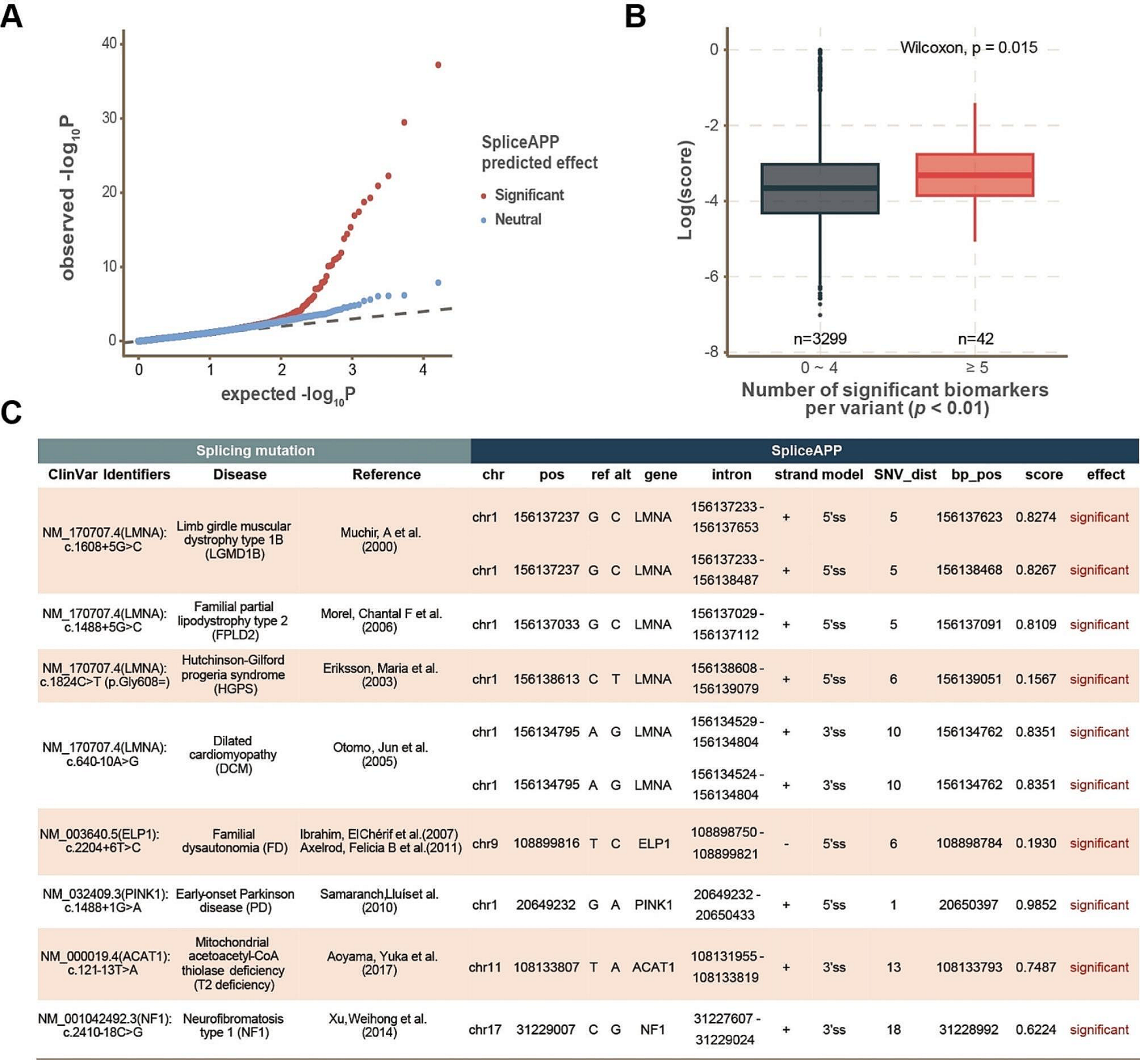
SpliceAPP prediction correlates with population health outcomes. **(A)** A quantile-quantile plot (Q-Q plot) shows an inflated distribution of p-values between SpliceAPP significant variants and population biomarkers in the Taiwan Biobank. Note that the variants collected by Taiwan Biobank have health-related implications, as evidenced by the skewed p-value distribution (both red and blue) from the theoretical p-value distribution (dashed lines). **(B)** Variants associated with more than five aberrant biochemical test values (*p* < 0.01) are predicted with higher splice variant scores by SpliceAPP. **(C)** Prediction outcome of known disease-related splicing mutations with SpliceAPP (19, 20 and the references therein)

We employed SpliceAPP to classify splicing variants within the Taiwan Biobank dataset, which comprises over 10^5^ genetic polymorphisms from 68,978 community samples featuring various health-related biochemical measurements and self-reported disease information. Among 3,341 single nucleotide polymorphisms (SNPs) with a frequency above 0.01 that were assessed by SpliceAPP, 335 were identified as splicing variants. To demonstrate the significance of these predictions, we evaluated the association of the 3,341 variants with 24 biochemical test values. Linear regression was used to assess the association between each SNP and continuous biochemical indices, while logistic regression was implemented to test the association between each marker and categorical traits. For comparability, we selected 335 variants predicted to be neutral with the lowest scores by SpliceAPP and minor allele frequencies (MAF) similar to those of the significant variants (± 0.01). The p-values calculated from these associations were used to generate a quantile-quantile plot (Q-Q plot) for both the 335 neutral variants and the 335 significant variants. By comparing the correlation between aberrant biochemical indices of significant splicing variants or neutral variants predicted by SpliceAPP, we observed a significant deviation in association p-values (indicated by inflation from the background in the Q-Q plot) for the SpliceAPP splicing variants **(**Fig. 6A**)**. Moreover, variants associated with more than five aberrant health indices showed higher splice variant scores as calculated by SpliceAPP **(**Fig. 6B**)**. These results suggest that SpliceAPP effectively identifies splicing defects relevant to health outcomes in the population. Additionally, we used SpliceAPP to predict reported splicing mutations and demonstrated that these mutations significantly impact splicing outcomes **(**Fig. 6C**)**, consistent with clinical reports [19, 20]. This consistency reinforces SpliceAPP’s effectiveness in evaluating the impact of genetic variants on splicing and their associated health outcomes, confirming its potential as a robust method for clinical and population health research.

Notably, transcripts harboring splicing variants were predicted with a significant loss of the gene product, potentially attributed to the nonsense-mediated mRNA decay mechanism. In instances where truncated proteins or proteins with missing domains were produced, we observed alterations in signaling transduction, leading to abnormal downstream regulation. These findings underscore the detrimental impact of splicing variants, emphasizing the importance of identifying and understanding their effects.

We acknowledge the significance of tissue-specific regulation in splicing, which may play a crucial role in splicing decisions in the context of intronic mutations. However, SpliceAPP has not identified tissue-specific factors underlying splicing decisions. All the features considered are characteristics of the pre-mRNA, such as evolutionary conservation, the distance between the variant and the splice site, and the strength of the splice sites. Consequently, SpliceAPP’s predictions are not tissue-specific.

## Conclusions

SpliceAPP focuses on predicting splicing errors of near-exon intronic sequence variations, aiming to fill a gap in intronic variant interpretation, given that several other efforts focus on exonic splicing variant prediction [10–12]. While SpliceAPP diagnoses variants in near-exon regions, where splicing variants are enriched, it does not predict deep intronic mutations. Overall, we demonstrated superior accuracy in splicing variant prediction over benchmarking tools and showcased its association with aberrant health phenotypes in the population. By annotating splicing errors and intronic splicing signals, we anticipate that SpliceAPP can accelerate the functional interpretation of genome variations.

## Availability and requirements

Project name: SpliceAPP.

Project home page: https://bc.imb.sinica.edu.tw/SpliceAPP/.

Operating system(s): platform independent.

Programming language: C++, PHP, HTML, CSS, and JavaScript.

Other requirements: web browsers, internet connectivity.

License: none.

Any restrictions to use by non-academics: none.

## Data Availability

The datasets analyzed during the current study are available at the NCBI GEO: GSE179892 and GSE120695.

## Abbreviations

5’ss: 5’ splice site
3’ss: 3’ splice site
snRNA: small nuclear RNA
snRNP: small nucleoproteins
AI: Artificial intelligence
bp: Basepairs
VCF: Variant Call Format
IGV: Interactive Genomics Viewer
